# Tactile Frequency-Specific High-Gamma Activities in Human Primary and Secondary Somatosensory Cortices

**DOI:** 10.1038/s41598-017-15767-x

**Published:** 2017-11-13

**Authors:** Seokyun Ryun, June Sic Kim, Hyeongrae Lee, Chun Kee Chung

**Affiliations:** 1 Interdisciplinary Program in Neuroscience, Seoul National University College of Natural Sciences, Seoul, 08826 Korea; 2 Department of Brain & Cognitive Sciences, Seoul National University College of Natural Sciences, Seoul, 08826 Korea; 3 Department of Mental Health Research, National Center for Mental Health, Seoul, 04933 Korea; 4 Department of Neurosurgery, Seoul National University College of Medicine, Seoul, 03080 Korea

## Abstract

Humans can easily detect vibrotactile stimuli up to several hundred hertz, but underlying large-scale neuronal processing mechanisms in the cortex are largely unknown. Here, we investigated the macroscopic neural correlates of various vibrotactile stimuli including artificial and naturalistic ones in human primary and secondary somatosensory cortices (S1 and S2, respectively) using electrocorticography (ECoG). We found that tactile frequency-specific high-gamma (HG, 50–140 Hz) activities are seen in both S1 and S2 with different temporal dynamics during vibration (>100 Hz). Stimulus-evoked S1 HG power, which exhibited short-delayed peaks (50–100 ms), was attenuated more quickly in vibration than in flutter (<50 Hz), and their attenuation patterns were frequency-specific within vibration range. In contrast, S2 HG power, which was activated much later than that of S1 (150–250 ms), strikingly increased with increasing stimulus frequencies in vibration range, and their changes were much greater than those in S1. Furthermore, these S1-S2 HG patterns were preserved in naturalistic stimuli such as coarse/fine textures. Our results provide persuasive evidence that S2 is critically involved in neural processing for high-frequency vibrotaction. Therefore, we propose that S1-S2 neuronal co-operation is crucial for full-range, complex vibrotactile perception in human.

## Introduction

Our ability to detect vibrations through glabrous skin has important roles in everyday tactile experiences. We can acquire information about the surface geometry of objects^1^, the roughness of fine textures^2^ and specific vibrotactile frequencies^3^ through this. Although it is largely unknown how our brain perceives various vibrotactile stimuli differently, a common belief for this issue is that stimulus-specific activity patterns of cortical neurons might be related to various vibrotactile perceptions^4,5^. At the somatosensory periphery, vibrotactile information is mainly encoded by two predominant vibration-sensitive receptors: Meissner (most sensitive in the flutter range, 5–50 Hz) and Pacinian (most sensitive in the vibration range, 100–400 Hz) corpuscles^6^. Since both have distinct sensitive frequency ranges, it has traditionally been considered that neural processing of these frequency ranges might have different mechanisms at the cortex^6^.

As a major entrance of the somatosensory information, S1 has a crucial role in the early-stage vibrotactile processing. Previous micro-level animal studies have suggested that the flutter frequency is mainly encoded in the firing rate based patterns^3,7^, whereas the vibration frequency is encoded in millisecond-precision spike timing^1,8^. Although these micro-level studies have given some important insight, it is still unknown how the information from these S1 neurons contributes to the specific vibrotactile perception including flutter and vibration in human. This is challenging work in micro-level studies because millions of neurons in various brain areas are involved in vibrotactile processing. Indeed, at the population level, a recent stereo-EEG study has indicated that more than 10% of cortical surface exhibits significant HG activation even in single-pulse median nerve stimulation^9^. In light of this, it is possible that a specific vibrotactile perception might be built up across a large-scale cortical co-operation including S1, S2 and other sensorimotor-related areas. Specifically, S2 is regarded as one of the key areas of such mechanism because population activities of S2 sometimes exhibited vibration dependency. For example, several human neuroimaging studies have shown the difference in blood oxygen level-dependent (BOLD) activities among various frequencies as well as between flutter and vibration in both S1 and S2^10–12^. However, these results are inconsistent, and no electrophysiological evidence related to them has been reported. Furthermore, it remains largely unknown what components of the S1 and S2 neuronal population activities directly represent various stimulus frequencies including the flutter and vibration. Finally, an important question regarding these notions is how the human brain processes the wide-frequency range of vibrotactile information with different neuronal spatio-temporal dynamics.

Tactile stimuli from the scanning of textured surfaces elicit complex vibration patterns, and the composition of their frequencies is mainly determined by the surface microgeometry of the texture^13^. Recent studies have shown that the activation patterns of cutaneous mechanoreceptive afferents such as Meissner and Pacinian fibers are closely related to texture-elicited vibrotactile frequencies^14,15^. However, it is unclear how these complex activation patterns are represented at the cortex. Furthermore, to our knowledge, it has never been reported whether the neuronal responses to texture stimuli can be explained by those of simplified artificial ones.

To address these issues, we recorded human ECoG data in several regions including the S1 and S2 during flutter and vibration stimulation. First, we aimed to investigate whether S1 and S2 show different neuronal population activities when pin-point flutter and vibration stimuli are delivered. Second, we tested whether these activities show consistent frequency-dependent changes in various frequencies including flutter and vibration. Finally, we investigated the possibility that the neural activity patterns seen in pin-point stimulation can also be observed in texture stimuli.

## Results

### S1/S2 HG Activities during Vibrotactile Stimuli

**Figure 1:**
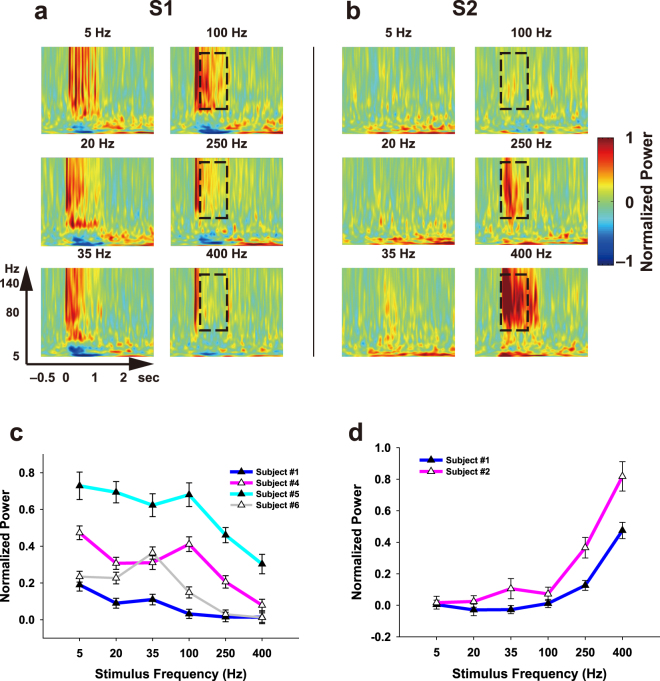
Representative time-frequency plots for various stimulus conditions (5, 20 and 35 Hz for flutter; 100, 250 and 400 Hz for vibration) in S1 (**a**), from Subject #4) and S2 (**b**), from Subject #2). Time t = 0 and t = 1 s indicate stimulus onset and offset, respectively. Dashed boxes indicate the time (0.2 to 0.9 s after stimulus onset) and the frequency range (50 to 140 Hz) which shows prominent HG power decreases in S1 (**a**), and increases in S2 (**b**) with an increase the stimulus frequencies above 100 Hz (**c** and **d**). Line plots for HG power levels from Subjects #1 (blue), #4 (pink), #5 (cyan) and #6 (gray) in S1 (**c**) and from Subjects #1 (blue) and #2 (pink) in S2 (**d**). Error bars in both *C* and *D* denote the s.e.m. Significance testing results among the various stimulus conditions are shown in Table S1.

To test whether neuronal population signals from the ECoG change depending on the flutter (5, 20 and 35 Hz) and vibration (100, 250 and 400 Hz) stimuli, we first performed time-frequency analysis for each stimulus condition. We found distinct HG power changes both in S1 and S2 during flutter and vibration stimuli, and their patterns were highly frequency-specific in vibration conditions. Furthermore, their frequency-dependent decreasing/increasing patterns during vibration stimulation were clearly different between the S1 and S2 areas (Fig. 1, Supplementary Fig. S1). Consistent with a previous finding, we observed significant HG activity in the premotor area during the stimulation, but whether it depended on the stimulus frequency was unclear^9^.

In S1, dominant HG responses ranging from 50 to 140 Hz were found during all stimulus periods. These responses started to increase at 27 ± 10 ms (mean ± standard deviation) after stimulus onset and lasted until stimulus offset. No significant difference was found between the onset timings of the S1 HG activities and the event-related potentials (ERPs) (paired *t*-test; *t* = 1.60, *P* = 0.13). At the flutter range, the power differences among the three conditions were relatively small and showed inconsistent patterns (Fig. 1c, Supplementary Table S1). These trends were also seen at the 100 Hz vibration frequency; however, the HG power significantly decreased when increasing the stimulus frequency above 100 Hz. Consistent decreasing patterns in the HG power under the vibration condition were observed in all four subjects (Fig. 1c). The S1 HG data from Subject #1 was excluded from subsequent analysis because its signal-to-noise ratio was extremely low.

In S2, there were no distinct HG responses under the flutter conditions. Notably, however, its power strongly increased with increasing stimulus frequencies under the vibration conditions (>100 Hz). Moreover, the HG power for vibration in S2 was more strikingly changed than those in S1. These increasing patterns were consistently seen in both subjects (Fig. 1d). The HG responses formed maximum peaks at 150–250 ms after stimulus onset and lasted 250–300 ms after stimulus offset. The power differences among the vibration conditions were highly significant (Table S1), while the powers among flutter conditions were not statistically different. Interestingly, although the 100 Hz stimulus frequency has conventionally been considered the vibration range, the HG power of this was almost the same as those of the flutter conditions, and these trends were analogous to the results in S1. We also observed similar patterns from Subject #1’s intracerebral depth electrodes inserted close to the S2 area (Fig. S3).

We performed the same analysis in the theta (4–7 Hz), alpha (8–14 Hz), beta (15–30 Hz) and low-gamma (30–50 Hz) bands. No consistent and significant differences across the subjects and conditions were found except the low-gamma band. The changes in low-gamma activities might be due to the power leakage from the HG band. Thus, their relative power differences were smaller than those of the HG band. We also investigated S1/S2 ERP patterns under the vibration conditions. No distinct ERP peak patterns showing frequency dependency, were detected in S1. However, strong and long-latency (150–250 ms after stimulus onset) S2 peaks were observed in the 400 Hz stimulus conditions (Fig. S4). They seemed to be closely related to the S2 HG power because the latencies and increasing patterns of these ERP peaks were similar to those of the HG peaks in S2.

Next, we confirmed that these HG power differences were only detected in S1 and S2. To do this, we extracted the HG power from all the electrodes during the flutter and vibration stimuli and then tested their differences (Fig. S2). The results show that prominent HG power differences are found in S2 (the posterior part of the upper bank of the Sylvian fissure) and S1.

### S1 HG Attenuation during Flutter and Vibration

**Figure 2:**
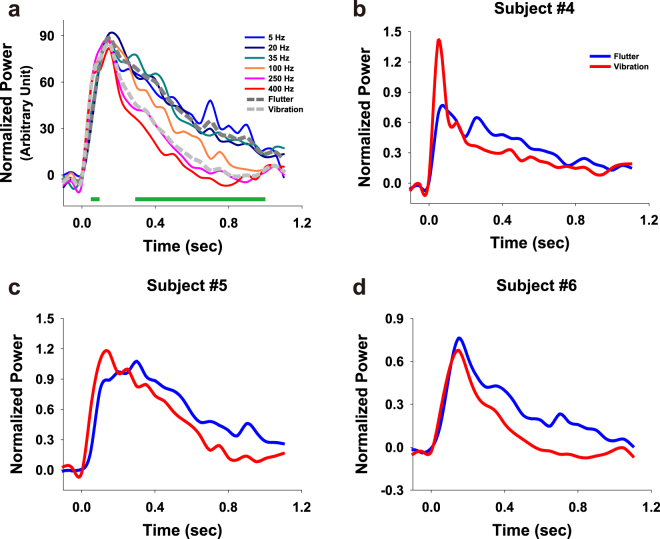
S1 HG power time series during the flutter and vibration stimuli. Data were binned for statistical testing and smoothed for visualization. Time t = 0 s indicates stimulus onset. (**a**) Average power time series plots across all three subjects (Subjects #4, #5 and #6). Each line indicates the 5 (blue), 20 (dark blue), 35 (dark cyan), 100 (orange), 250 (pink) and 400 Hz (red) conditions. Dashed dark gray and gray lines denote the average flutter and vibration conditions, respectively. The green bars indicate the time bins which show significant power differences between the flutter and vibration conditions (P < 0.001, Bonferroni corrected). (**b**–**d**) HG power time series of individual subjects. The blue and red lines show the flutter and vibration conditions, respectively.

In the previous section, we showed that the S1 HG power during stimulus periods decrease with increasing stimulus frequencies under vibration conditions. However, in terms of temporal dynamics, the power of the HG peaks, which is formed at the early stage of the stimulus periods (50–100 ms after stimulus onset), showed no distinct difference between the flutter and vibration conditions in Subjects #5 and #6. Moreover, the power of the peaks in vibration was sometimes higher than those in flutter (Fig. 2b–d). Therefore, we hypothesized that these power differences in S1 are due to different amounts of temporal decrease in the HG power between the flutter and vibration conditions. To assess this question, we first calculated binned HG power time series for each stimulus condition. Because the maximum peak powers were different among the stimulus conditions and also among the subjects, we divided these peak powers into the power time series data for normalization. HG power in flutter showed more sustained activity during the stimulus period than that in vibration. However, the power in vibration was quickly attenuated (Fig. 2). The differences in power decreases between the flutter and vibration were significant across most of the time bins after the maximum peak (independent two-sample *t* test, *P* < 0.001, Bonferroni corrected). Importantly, the attenuation of the HG power in vibration showed a stimulus frequency-specific pattern, while no difference was found among the flutter conditions (Fig. 2a).

### Single-Trial Vibration Frequency Classification

**Figure 3:**
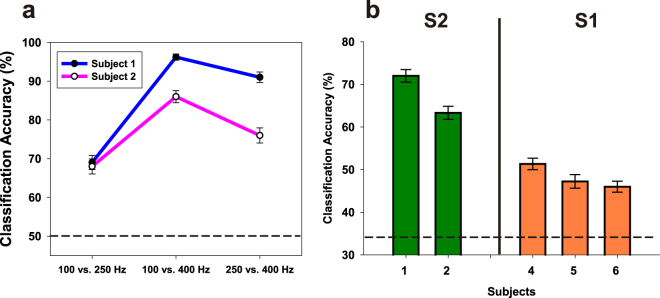
(**a**) The accuracy of the stimulus frequency classification by simple SVM (S2 only). The x axis indicates three possible stimulation pairs. The chance level is 50% (dashed line). (**b**) The accuracy of the stimulus frequency classification by multiclass SVM using the single-trial HG power in S2 (left, green bars) and S1 (right, orange bars). The dashed line indicates the chance level (33%). Error bars in both (**a**) and (**b**) indicate the s.e.m.

Our quantitative results indicate that HG power changes in the S2 area during vibration stimuli are more dominant than those in S1, and their frequency-specific patterns are highly significant. Taking this result into consideration, we assumed that if the HG powers in S2 show consistent frequency-dependent changes across all trials under vibration conditions, the frequencies can be easily discriminated by the stimulus-related HG power from single-trial ECoG data. To address this question, we extracted features from single-trial HG power with two or three electrodes, and then we calculated the classification accuracy with the simple and multiclass linear support vector machine (SVM). We first estimated condition-by-condition classification accuracy using a simple SVM to compare the relative performance among three possible condition pairs. As expected, all condition pairs from Subjects #1 and #2 can be classified with high accuracy ranging from 69.0–96.2% (chance level = 50%), and the highest performance was achieved with the 100 vs. 400 Hz condition (96.2% from Subject #1, 86.0% from Subject #2) (Fig. 3a). Classification accuracies over the three stimulus conditions by multiclass SVM were 72.0 and 63.3% (chance level = 33%) in Subjects #1 and #2, respectively (Fig. 3b, left). We also evaluated the classification performance using the S1 HG data. Their accuracies were above the chance level but lower than those in S2 (Fig. 3b, right).

### S1/S2 HG Activities during Texture Stimuli

**Figure 4:**
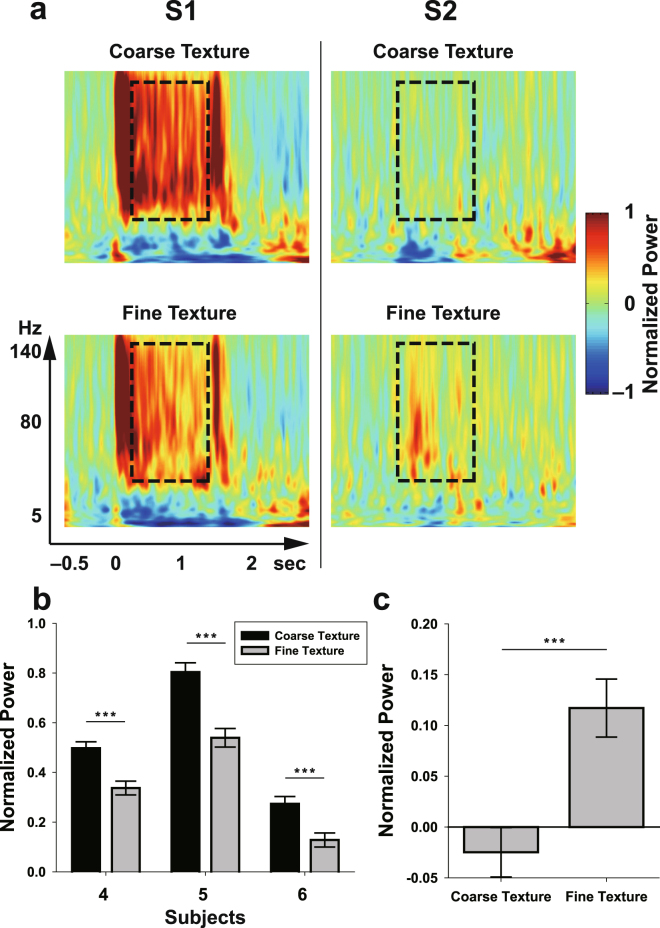
(**a**) Representative time-frequency plots for coarse (top) and fine (bottom) textures in S1 (left column, from Subject #5) and S2 (right column, from Subject #3). Time t = 0 and t = 1.5 s indicate stimulus onset and offset, respectively. Dashed boxes indicate the time (0.2 to 1.3 s after stimulus onset) and frequency range (50 to 140 Hz). Bar plots denote the HG power levels in S1 (**b**) and S2 (**c**, from subject 3) during coarse and fine texture stimulations. Error bars indicate the s.e.m. The power differences between the two conditions were significant across all subjects (*P* < 0.001).

Although our results from the pin-point vibrotactile stimulation showed vibration-specific neural activities which are distinguished from the neural activities in flutter, it is unclear whether these results can be applied to a natural environment such as surface scanning for natural textures through the glabrous skin. To assess this issue, we calculated the HG powers in S1 and S2 during coarse and fine texture stimuli (Fig. S5). Although a previous study indicated that the firing rates of Pacinian (PC) afferents at the peripheral level are high in both coarse and fine texture stimulations^14^, the relative activation ratios between rapidly adapting type 1 (RA or Meissner) and PC afferent populations in coarse and fine texture stimulations might be similar to those in our pin-point flutter and vibration stimulations. Interestingly, we observed similar HG power decreasing/increasing patterns in S1 and S2 to those from the pin-point vibrotactile experiment (Fig. 4a). In S1, although there may exist an additional effect in the coarse texture condition due to the neuronal activity of slowly adapting type 1 (SA1) afferents, HG power of the coarse texture stimulation was stronger than those of the fine texture stimulation across all three subjects (Fig. 4b). Notably, in contrast to the HG patterns in S1, a significant S2 HG power increase was observed in the fine texture condition compared to the coarse one (Fig. 4c). Its temporal pattern was similar to the results from pin-point stimuli (200–300 ms after stimulus onset). Furthermore, these S1/S2 power differences between the coarse and fine textures were consistently observed regardless of the scanning direction (two-way ANOVA; interactions: texture × direction; *F*_(1, 156)_ = 0.83, *P* = 0.36 in Subject #3; *F*_(1, 156)_ = 0.02, *P* = 0.89 in Subject #4; *F*_(1, 156)_ = 0.73, *P* = 0.40 in Subject #5; *F*_(1, 316)_ = 0.07, *P* = 0.79 in Subject #6). Note that a distinct HG power increase in the fine texture stimulation in comparison to that in the coarse texture stimulation was only observed in the S2 area (Fig. S6).

## Discussion

Previous studies such as those involving single unit recording and optical intrinsic signal (OIS) imaging in animals reported that there were prominent decreases in the mean rates of neuronal firing and absorbance during 200 Hz vibration stimuli as compared to 20 Hz flutter stimuli in S1^16,17^. The literature also shows that the firing rates of the initial stimulation period (100 to 300 ms) were nearly identical in both conditions, but then declined rapidly in the vibration condition. Our findings with regard to the S1 HG attenuation pattern during flutter and vibration stimuli are very similar to the results of these earlier studies. Indeed, recent studies have indicated that the temporal evolution associated with local field potential (LFP) HG activities are tightly correlated with the time-series of the average firing rate^18,19^. In light of this, although not all HG activity stems from neuronal spiking activity^20^, it is possible that we found the same patterns of neuronal responses at the macroscopic level in humans.

In the present study, frequency-dependent attenuation patterns of HG activities in S1 were also found within the vibration range whereas these patterns were virtually identical within the flutter range. If the HG power is closely related to the firing rates of the neuronal population^18,19^, our results in S1 can be explained in relation to the evidence of micro-level studies. Although HG activities only represent a small portion of the neural activities in S1, our result may stem from 1) the differences in the population and frequency sensitivity between the RA and PC neurons in S1, or 2) the stimulus frequency-dependent adaptation property of the S1 neuronal population. In fact, RA neurons are densely distributed in the S1 area, while the population of PC neurons is relatively sparse^21,22^; hence, the population activities of RA neurons may more strongly affect the HG power than those of PC neurons. Because the sensitivity of RA neurons gradually decreases with increasing stimulus frequencies in the vibration range^23^, the overall amount of afferent neuronal activity can be decreased with increasing stimulus frequency. In our results, however, the S1 HG peaks did not show frequency dependence. Thus, it may be reasonable to assume that most of the touch-sensitive afferent neurons in the S1 area tend to be highly activated at the first transient stimulus of the stimulus train^24^, after which they exhibit different adaptation properties depending on the stimulus frequency in the vibration range. Similarly, several studies reported that neuronal adaptation in the rat barrel cortex increases with increasing stimulus frequencies in the flutter range and that this adaptation property is related to the discrimination performance of the tactile frequency^5,25,26^. In this study, we could not observe this frequency-specific HG adaptation in the flutter range. However, it might be due to the fact that the mean firing rates of the S1 afferent neurons also increase with increasing stimulus frequencies in the flutter range^4^. Accordingly, it is possible for the overall amount of neuronal activation not to show any distinct differences among such frequencies. In contrast, the firing rate patterns of S1 afferent neurons are nearly independent in the vibration range^1^. Therefore, our results may indicate large-scale, frequency-dependent neural adaptation in the vibration range.

Our results may provide some insight into the ongoing debate about the serial versus parallel processing of S1 and S2 during vibrotactile stimulation^27,28^. Although we could not investigate the causal relationship between them, it is unlikely that the HG activation in S2 (the posterior part of the upper bank of the Sylvian fissure) directly represents the primary response for a high-frequency stimulus. For several reasons, we suggest that these activations depend on the signals from the upstream cortical area, such as S1, and that they may be determined by the interaction among different types of afferent neurons in S1. First, the S2 HG responses to vibration stimuli showed a relatively long latency (150–250 ms after stimulus onset) compared to the S1 HG responses (50–100 ms). Moreover, the S2 HG responses lasted 250–300 ms after stimulus offset. If S2 directly receives inputs from PC afferents, both the S1 and S2 HG responses should have similar latency and duration properties. This serial processing mechanism is strongly supported by the findings of many previous studies^29–31^, although we did not observe the neural activity in the entire S2 region. Second, the S2 HG patterns depending on the vibration frequencies did not match the frequency-dependent sensitivity patterns of the PC afferents (most sensitive around 200–250 Hz)^6,32^. These results indicate that the S2 HG responses do not reflect the early stage of vibrotactile processing and that neuronal interactions in the high-frequency vibration should occur at upstream regions such as S1 and other parietal areas. Third, in the texture stimulation, strong HG power was observed only in the fine texture condition. As mentioned earlier, PC afferents are robustly activated by both coarse and fine textures^14^. If the HG activities in S2 exclusively show PC afferent dependency, they should be observed both in coarse and fine textures. Therefore, these results are explained if assuming hierarchical processing from S1 to S2 and the existence of some interaction between PC afferents and other afferents, such as RA afferents in S1, during vibrotactile processing. Indeed, several studies have indicated the existence of these types of interactions, or submodality convergences in S1^33,34^. Specifically, given the increased/decreased similarities between S1 and S2 HG activities, it is possible that the vibration-specific S2 HG patterns are determined by the relative proportion of the activations of RA and PC afferent neuronal populations in S1.

In this study, we found that the S1/S2, especially the S2, neuronal activation patterns for a tactile stimulus which contains dominant high-frequency vibration components show some degree of similarity between artificial pin-point and natural texture stimulations. This result suggests that although these two tactile experiences are perceptually different in terms of the stimulus type, the fundamental mechanisms of the S1/S2 neural processing of their associated vibratactile information are similar at the population level. Specifically, in terms of the functional role of S2 HG activity, this delayed and long-lasting activity may be partially related to neural processing for perceptual categorization, such as that related to roughness and smoothness. Although we could not investigate the perceptual relevance of the HG activity in S2, previous studies have indicated that neuronal responses in S2 encode tactile roughness, and this area is most likely involved in texture perception^35,36^. However, it is possible that we only observed a particular case of S2 responses for texture stimulation because texture stimuli contain not only vibratory information but also other types of information, such as spatial codes for shape perception^14,37^, and because we used only two texture stimuli for comparison. Therefore, further investigation is required to confirm this suggestion directly.

There are few subdural ECoG or EEG studies of human S2 because most of the S2 areas are located within the Sylvian fissures^38^. However, several macroscopic electrophysiological studies have indicated that particular somatosensory ERP in the peri-Sylvian areas represent S2 activities^39,40^. Importantly, a human study using stereo-EEG confirmed that the long-latency peri-Sylvian somatosensory ERP stems from S2 neural activations^41^. Furthermore, a recent stereo-EEG study reported a slowly responding HG pattern which lasted more than 200 ms after stimulus offset in the parietal operculum (S2) during median nerve stimulation^9^. In this study, we observed not only these ERP responses but also HG activities in the same area. Therefore, although we could not directly confirm the origin of these signals, previous findings and our results indicate that the vibrotactile-evoked HG signals from this area, especially the posterior part of the upper limb of the Sylvian fissure, represent frequency-specific S2 responses.

In the present study, we focused on neural activities depending on the frequency components of tactile stimuli. However, their amplitude dependency should be elucidated because the HG activities can also be modulated by stimulus amplitudes^19,42^. Specifically, further studies using complex waveform stimuli with various amplitudes are required to confirm our suggestion that S2 HG patterns are determined by the relative proportion of RA and PC afferent neuronal activations. Additionally, a possible limitation of our approach is the accuracy of the latency of the HG activity due to the limited temporal resolution of wavelets we used.

Our findings provide some insights into the functional roles of S2 in vibration processing. For example, it will be of interest to determine whether these S2 responses exhibit perceptual relevance. Further, the results for S2 can be applied to design a direct brain stimulation paradigm for generating or modulating high-frequency vibration sensations.

## Materials and Methods

### Subjects

Six patients with intractable epilepsy participated in this study. Patients underwent implantation of subdural electrode grids to monitor electrocorticograms. The subdural ECoG electrodes (Ad-tech Medical Instrument) had diameters of 4 mm with an inter-electrode distance of 10 mm. In Subjects #1, #4 and #6, intracerebral depth electrodes were inserted for clinical purposes. ECoG electrodes covered S1 (Subjects #1, #4, #5 and #6) and S2 (Subjects #1, #2 and #3). Preoperative MR images and postoperative CT images were acquired for each subject. All experiments and study procedures were approved by the Institutional Review Board of Seoul National University Hospital (H-1203-028-400). All subjects provided written informed consent before their participation (see Table S2 for additional patient details).

### Apparatus

Pin-point, sinusoidal vibrotactile stimuli were delivered by a customized piezoelectric actuator (stripe actuator; APC International, Ltd.) which covers the 1–500 Hz frequency range (Fig. S4). The deflection amplitude was controlled by the input voltage. To stimulate a fingertip, a small plastic pin (0.75 mm in diameter) was mounted on the tip of the piezoelectric material, and the actuator was insulated by a plastic box to avoid electrical artifacts. Stimulus waveforms were generated by micro controller unit and amplified to operate the piezoelectric material. Paradigm control was done using custom-made software written in Python (Python Foundation).

For texture stimulation, we used a custom-made texture stimulator, driven by an ultrasonic motor (USR60 E3N; Shinsei Corp.) which was in this case a MR/MEG-compatible non-magnetic model. To avoid other electromagnetic artifacts, the main body and rotating disc of the stimulator were made of plastic. The rotation direction and speed were controlled by a micro controller unit which has embedded software written in C and a customized computer program written in Matlab (MathWorks). Texture stimuli were delivered via a rotating disc stimulator (diameter: 190 mm) which positions the subject’s index finger above the surface of the disc during the no-stimulus periods (Fig. S5b, top). Because the sliding speed can vary depending on the distance from the center of the disc, we adjusted the location of the subjects’ fingertips such that the distances would be identical, with their finger fixed by a plastic ring which was mounted on a hand rest. Two texture materials were attached to the upper part of the rotating disc, and their attachment sites were on opposite sides of each quadrant of the disc (Fig. S5b, bottom). For soft contact between the finger and texture material, the stimulus onset/offset location had a round-shaped hillock.

### Experimental Design

Throughout the experiment, the subjects were instructed to look at a fixation cross and not to torn their view to the stimulator to avoid stimulus onset/offset anticipation. Because neural activities can vary depending on the degree of tactile attention during the stimulus conditions^43,44^, the subjects were also asked to pay attention to all stimuli.

For the pin-point vibrotactile experiment, stimuli were delivered to the index fingertip contralateral to the implantation site. We used six different stimuli to apply the flutter (5, 20 and 35 Hz) and vibration (100, 250 and 400 Hz) frequencies, with 50 trials for each frequency. The stimulus amplitudes of the flutter (180 μm) and vibration (90 μm) were roughly adjusted to equalize the subjective intensity levels^21,32^. The stimulus period of each trial was 1 s, with an inter-stimulus interval of 2.5, 3, or 3.5 s, and all trials and inter-stimulus intervals were randomized. No stimulus-related auditory sound was detected throughout the experimental period.

For texture stimulation, two passive tactile stimuli were delivered to the index finger contralateral to the implantation site by the rotating texture disc with a normal force of 15–30 g wt., a sliding speed of 63 mm/s (60 mm from the center of the texture disc) and a rotating speed of 10 rpm. One stimulus was a 2 mm grid texture (coarse texture) and the other stimulus was a sandpaper-like fine texture (particle size <50 μm, non-periodic particle pattern) which was made of acrylonitrile butadiene styrene (Figs. S5c and 5d). The two textures were selected based on the results of previous studies^13–15^. These studies indicated that coarse texture stimuli highly activate SA1, RA and PC afferents, while fine texture stimuli robustly activate only PC afferents. We could not control the effect of SA1, which shows dominant activation from coarse textures because the firing patterns are very similar between SA1 and RA afferents across various texture conditions^14^. The expected peaks of the elicited frequencies in the fingertip by the coarse and fine texture stimulations were 30–35 Hz (flutter range) and 200–250 Hz (vibration range), respectively^13^. The stimulus period was 1.5 s, with an inter-stimulus interval of 3.5, 4, or 4.5 s. To avoid directional bias, each texture stimulus was delivered in a clockwise or counter-clockwise direction with 40 trials per direction (Fig. S5b, bottom). The rotation directions (clockwise/counter-clockwise) were randomly selected.

### Data Acquisition and Preprocessing

ECoG data were recorded with a 128-channel amplifier system (Neuroscan). Signals were digitized at 1000 (for Subject #1) or 2000 Hz and were band-pass filtered at 0.1–200 or 0.1–500 Hz, respectively. ECoG channels which show epileptiform activities and abnormal signals due to technical problems were excluded from further analysis. The recorded data were re-referenced to the common average reference (CAR). To remove systematic noise at 60 Hz and related harmonics, the signals were notch-filtered with a finite impulse response (FIR) filter using the eegfilt function in the EEGLAB toolbox. Epoching was performed with a window of –1 to 3 s of stimulus onset. For the localization of the ECoG electrodes, co-registrations between preoperative MRI and postoperative CT images were performed semi-automatically with the CURRY software (version 7.0; Compumedics Neuroscan).

### Analysis

All analyses were performed with Matlab. We initially determined which electrodes represent S1 and S2 neuronal activities. We chose S1 electrodes based on the anatomical landmark of the hand knob and on well-known S1 tactile responses, such as strong ERP responses which have a delay of 30 ± 15 (mean ± standard deviation) ms and event-related desynchronization (ERD) responses. S2 electrodes were chosen according to the findings of previous ECoG studies, which demonstrated that ECoG electrodes located on the upper limb of the Sylvian fissure in the parietal region show neural responses from S2^40,41,45^.

For a time-frequency analysis, the continuous Morlet wavelet transform was applied to the epoched data. In this analysis, the effective window length (95% confidence interval of the Gaussian kernel, seven cycles) was 80 ms at 50 Hz. Transformed single-trial data were squared for calculating power and then normalized by the mean and standard deviation of the baseline power (–1 to 0 s of stimulus onset) of each frequency. For visualization, the normalized single-trial data were temporally smoothed with a window of 50 ms and were then averaged across all trials. Note that the averaged data does not indicate the Z-score.

To test the significance among the various stimulus frequency conditions, the HG power of each stimulus condition was averaged across the frequency range of 50–140 Hz, and the stimulus period ranged from 0.2 s after stimulus onset to 0.1 s (0.2 s for texture) before stimulus offset (dashed line in Fig. 1). We did not include the transient peak periods of the S1 HG activities ranging from 50–100 ms after stimulus onset because their power outcomes showed inconsistent patterns among the frequency conditions. However, the overall results did not change when we included these periods. We initially determined the HG range based on previous studies^9,18,46^. However, we found that the boundaries of the HG band showed marked inter-individual differences. Thus, we adjusted the bandwidth finely by undertaking a visual inspection of our dataset (50–140 Hz). To determine the on/offset times of the HG activities, the averaged HG trace was normalized by the mean and standard deviation of the baseline power, after which the start/end of time points which exceeded two standard deviations were defined as the HG onset/offset^47,48^. Independent two-sample *t* test and one-way ANOVA were performed for significance testing (Table S1). Before significance testing, we used the Lilliefors test for normality testing (*P* > 0.01, for all datasets). For multiple comparisons among the HG power levels at various frequencies, we used the Bonferroni correction procedure.

To obtain brain maps pertaining to HG power differences between the flutter (coarse) and vibration (fine) conditions, we performed the same procedure described above for all electrodes and extracted *t* scores with the independent two-sample *t* test.

To investigate the temporal decrease of the HG power during the stimulus periods, we initially calculated the single-trial power time series of this frequency range from each stimulus condition with the normalized time-frequency data above. The time series data were binned into 50 ms intervals with a time range from –0.4 to 1 s of stimulus onset, and the power data within each bin were averaged. For visualization, these binned single-trial time series were averaged across all trials, and spline smoothing was applied to the averaged time series. To obtain a grand average of binned time series data from the three S1 subjects, the data from each subject was normalized by the maximum peak amplitude of the averaged time series because the overall power of each subject was different. Significance testing for the HG difference between the flutter and vibration at each time bin was done with the independent two-sample *t* test, and their *P* values were corrected by the Bonferroni procedure.

For classification among the three vibration frequency conditions, first we extracted features from the single-trial HG power (the dashed line in Fig. 1) of two or three electrodes located on S1 or S2. To confirm whether the features based on the S1 or S2 HG power difference in the single-trial conditions represent their respective stimulus frequencies, we performed simple and multiclass linear support vector machine analyses. Classification performances were evaluated with five-fold cross validation.

## Electronic supplementary material

Supplementary Information
