# Supplementary material for: Tactile Frequency-Specific High-Gamma Activities in Human Primary and Secondary Somatosensory Cortices
Source: Sci Rep. 2017 Nov 13;7:15442. doi: 10.1038/s41598-017-15767-x (PMC5684355; doi:10.1038/s41598-017-15767-x)
Supplement: Supplementary file 1 — Supplementary Information [file 41598_2017_15767_MOESM1_ESM.pdf]

Supplementary Information

Title: Tactile Frequency-Specific High-Gamma Activities in Human Primary and Secondary Somatosensory Cortices

Author names and affiliations

Seokyun Ryun [1], June Sic Kim [2 *], Hyeongrae Lee [3] and Chun Kee Chung [1, 2, 4 *]

[1] *Interdisciplinary Program in Neuroscience, Seoul National University College of Natural Sciences, Seoul 08826, Korea*

[2] *Department of Brain & Cognitive Sciences, Seoul National University College of Natural Sciences, Seoul 08826, Korea*

[3] *Department of Mental Health Research, National Center for Mental Health, Seoul 04933, Korea*

[4] *Department of Neurosurgery, Seoul National University College of Medicine, Seoul 03080, Korea*

Correspondence should be addressed to Chun Kee Chung and June Sic Kim;

chungc@snu.ac.kr and jskim@hbf.re.kr

* Co-corresponding authors.

Chun Kee Chung, M.D., Ph.D.

Department of Neurosurgery, Seoul National University College of Medicine

103 Daehak-ro, Jongno-gu, Seoul 03080, South Korea

E-mail: chungc@snu.ac.kr

Telephone: +82-2-2072-2352

Fax: +82-2-744-8459

June Sic Kim, Ph.D.

Human Brain Function Laboratory, Seoul National University College of Medicine

17-408B, 103 Daehak-ro, Jongno-gu, Seoul 03080, South Korea

E-mail: jskim@hbf.re.kr

Telephone: +82-2-747-7271

Supplementary Figures

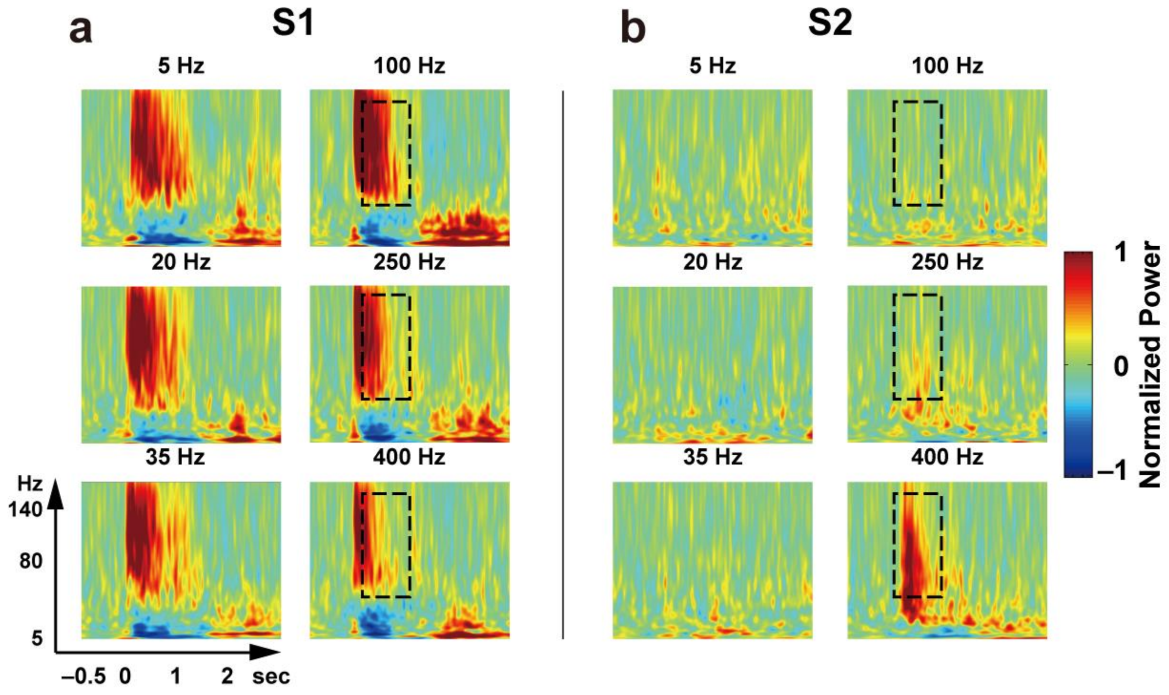

Figure S1. HG time-frequency results of other subjects. Time-frequency plots for various vibrotactile stimulus conditions. Legends and dimensions are the same as Fig. 1a and b. (a) S1 HG results from Subject #5. (b) S2 HG results from Subject #1.

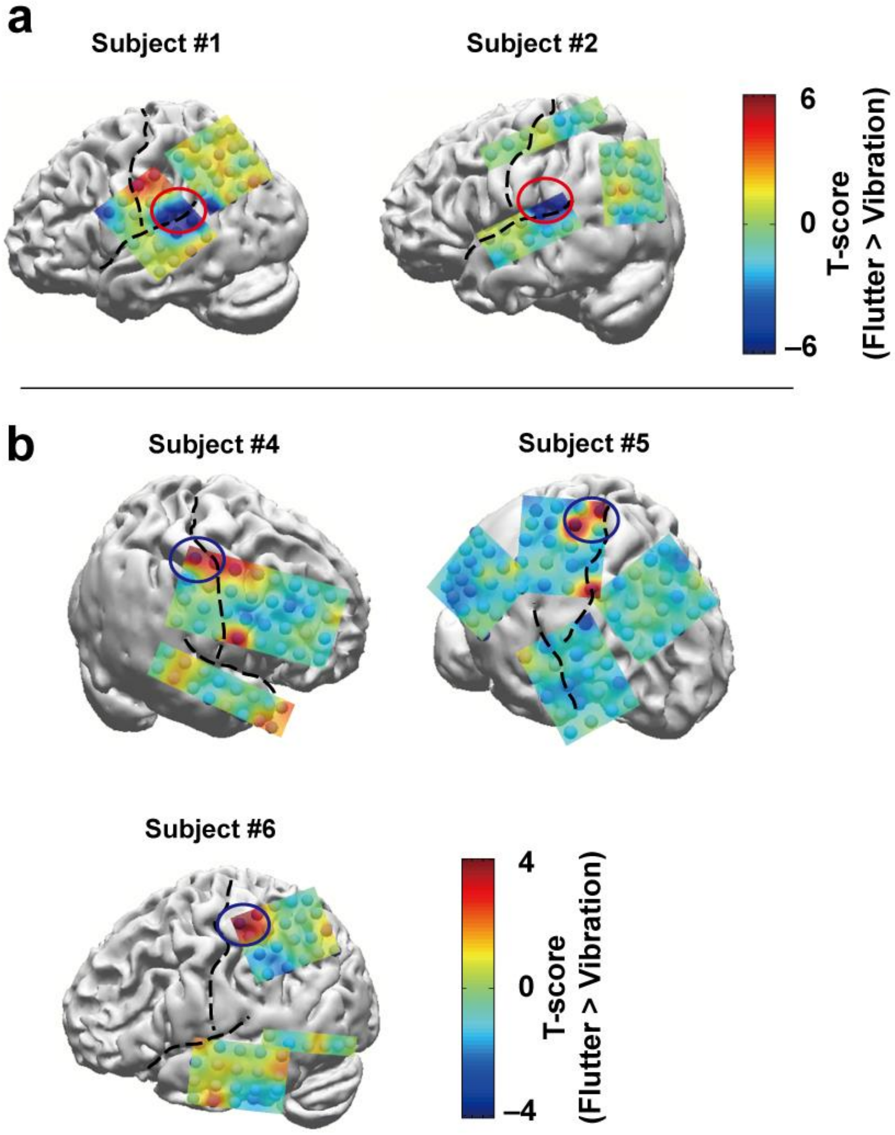

Figure S2. Topographical maps of the HG power differences between the flutter and vibration frequency stimulus conditions. Dashed lines indicate the central sulcus and Sylvian fissure. The blue and red circles indicate the S1 hand and S2 area, respectively. The red/blue areas in the topographical map indicate that the HG powers in the flutter conditions are higher/lower than those in the vibration conditions. (a) Topographical maps of Subjects #1 and #2. Both subjects show a dominant power difference in the same region, S2. In Subject #1, a power difference is also seen in the S1 region (red area). (b) Topographical maps of Subjects #4–6. These subjects' electrodes are located in the S1 region. Some electrode grids/strips were excluded because they were located at invisible sites.

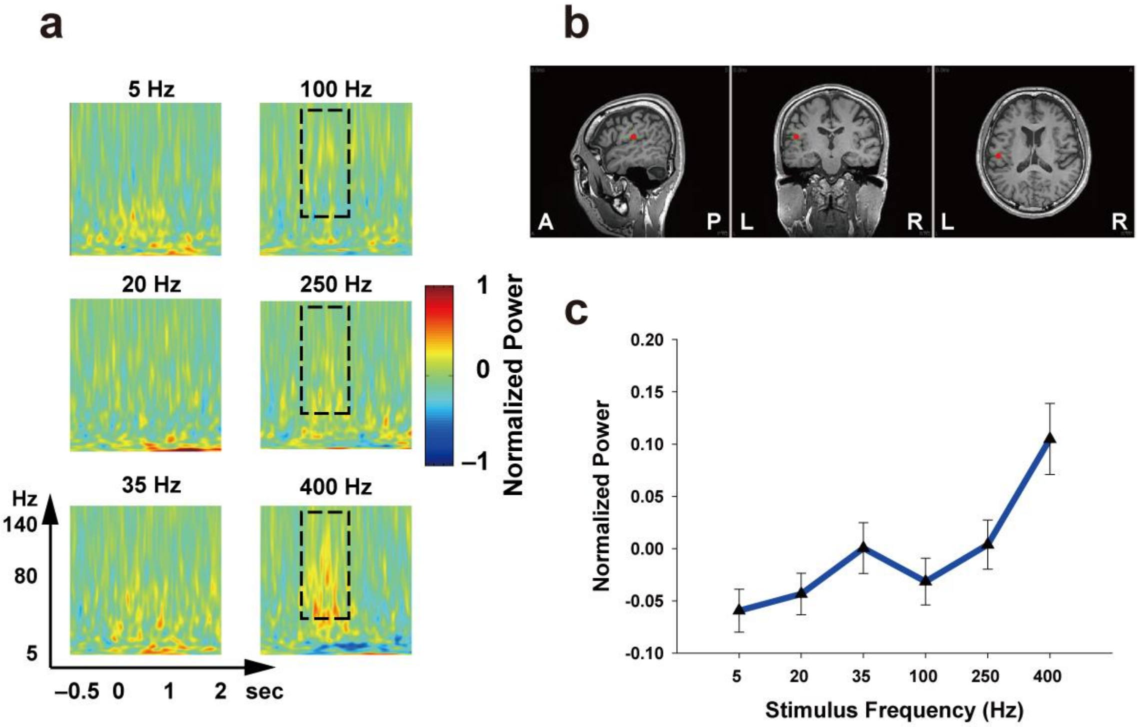

Figure S3. HG difference in depth electrode. (a) Time-frequency plots for various vibrotactile frequencies from Subject #1's depth electrode. (b) Location of the depth electrode. The depth electrode contact was located at the parietal operculum. (c) Line plots of the HG powers among various stimulus frequencies. Their powers were not strong but the patterns were analogous to the results from the ECoG electrodes. Error bars indicate s.e.m.

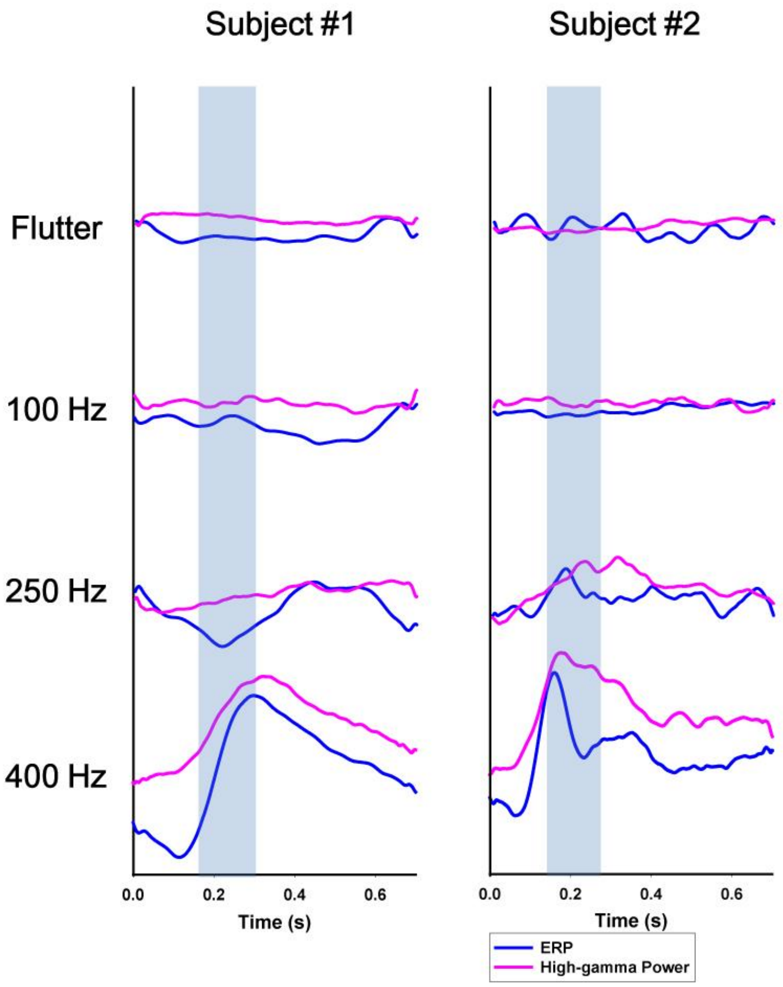

Figure S4. HG and ERP time-series in S2. S2 HG time series (pink) and simultaneous ERP (blue) at various vibrotactile frequencies in Subjects #1 and #2. Y-axes are arbitrary units for matching the scale between them. The results show that their peak patterns in the high-frequency conditions have similar timescales.

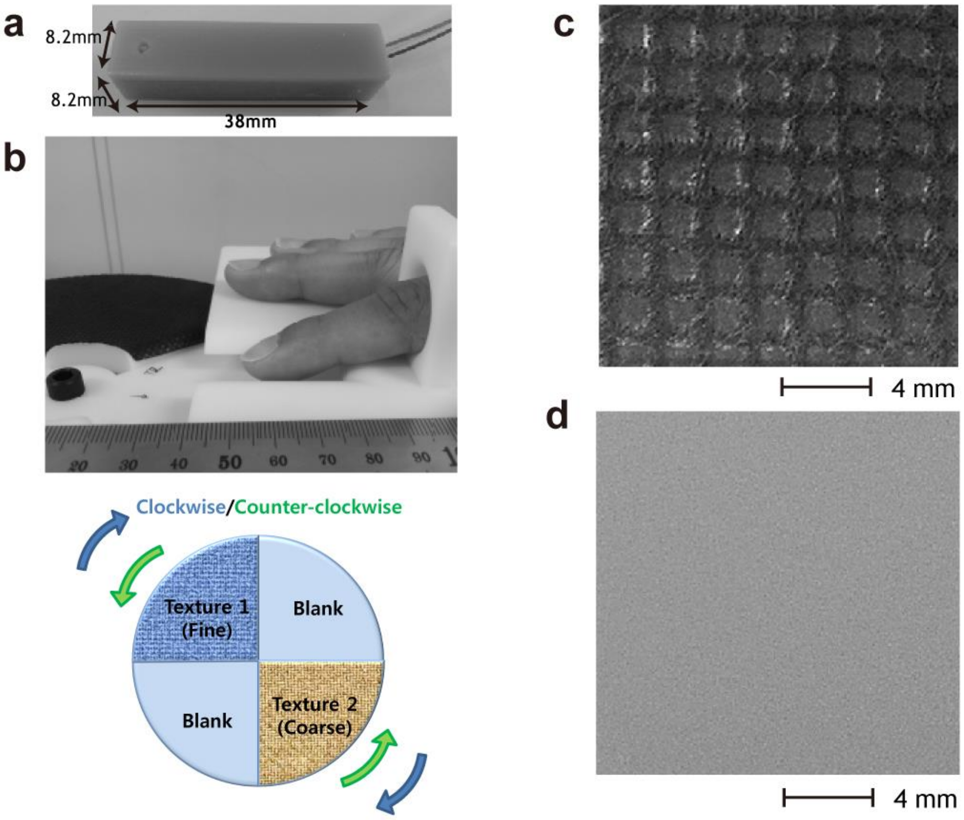

Figure S5. Apparatus and texture surface. (a) Pin-point vibrotactile stimulator. (b) Texture stimulator (top) and stimulation paradigm (bottom). No stimulus is delivered during blank periods. (c) Surface of the 2 mm grid texture for stimulating flutter frequency. (d) Surface of the fine texture for stimulating vibration frequency

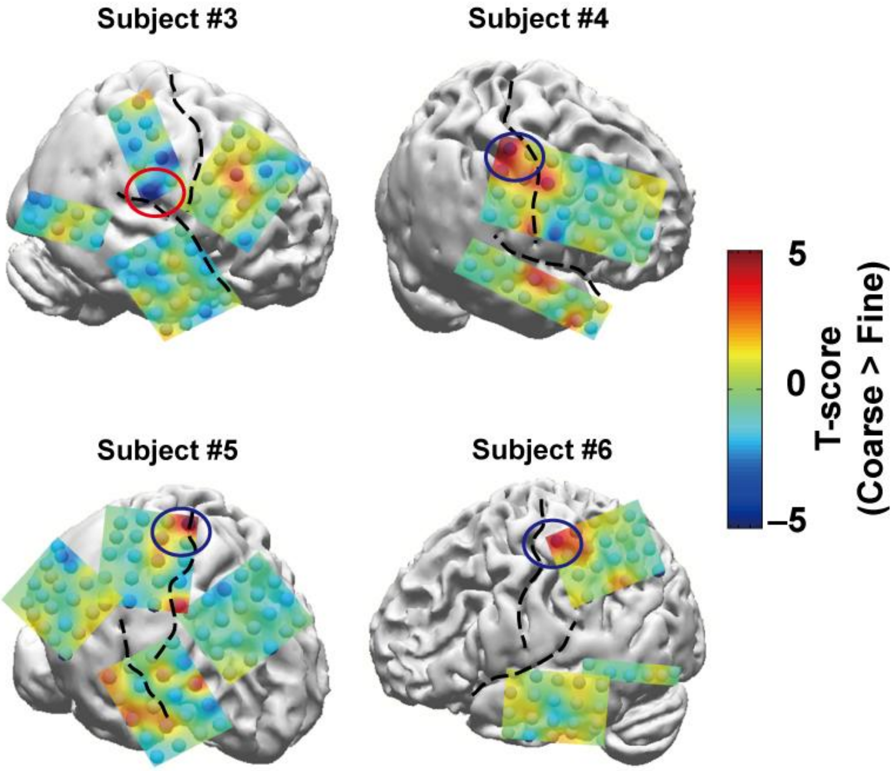

Figure S6. Topographical maps of HG power differences between the coarse and fine texture conditions. Dashed lines indicate the central sulcus or Sylvian fissure. The blue and red circles indicate the S1 hand and S2 area, respectively. The red/blue areas in the topographical map indicate that the HG powers in the coarse texture conditions are higher/lower than those in the fine texture conditions.

Supplementary Tables

Table S1. Results of significance testing among various stimulus frequencies

Subject (S1/S2)	Within flutter	Flutter vs. 100 Hz	Flutter vs. 250 Hz	Flutter vs. 400 Hz	100 vs. 250 Hz	250 vs. 400 Hz	100 vs. 400 Hz
#1 (S2)	0.69*	0.39*	5.57×10^{-4}	6.42×10^{-23}	0.049	8.94×10^{-7}	2.84×10^{-11}
	($F_{(2, 147)}$ = 0.38)	(t_{198} = 0.85)	(t_{198} = –4.03)	(t_{198} = –11.5)	(t_{98} = –2.76)	(t_{98} = –5.70)	(t_{98} = –7.90)
#2 (S2)	0.37*	0.67*	5.37×10^{-6}	4.62×10^{-20}	0.0023	0.0010	8.15×10^{-10}
	($F_{(2, 147)}$ = 1.01)	(t_{198} = 0.43)	(t_{198} = –5.11)	(t_{198} = –10.5)	(t_{98} = –3.72)	(t_{98} = –3.95)	(t_{98} = –7.21)
#4 (S1)	0.011	0.33	0.0020	1.1×10^{-9}	0.0014	0.057	3.80×10^{-8}
	($F_{(2,147)}$ = 6.84)	(t_{198} = 1.08)	(t_{198} = 3.70)	(t_{198} = 6.75)	(t_{98} = 3.88)	(t_{98} = 2.70)	(t_{98} = 6.40)
#5 (S1)	0.51*	0.98*	0.011	2.36×10^{-6}	0.032	0.13	1.19×10^{-4}
	($F_{(2, 147)}$ = 0.67)	(t_{197} = 0.02)	(t_{197} = 3.16)	(t_{197} = 5.21)	(t_{96} = 2.85)	(t_{96} = 2.32)	(t_{96} = 4.49)
#6 (S1)	0.021	0.0042	5.15×10^{-11}	4.11×10^{-12}	0.015	0.60*	0.0050
	($F_{(2,297)}$ = 5.94)	(t_{398} = 3.46)	(t_{398} = 7.06)	(t_{398} = 7.45)	(t_{198} = 3.12)	(t_{198} = 0.52)	(t_{198} = 3.45)

Unit = *P* values (Bonferroni corrected; * = uncorrected *P* values).

S1 = primary somatosensory cortex; S2 = secondary somatosensory cortex.

Table S2. Demographics of the subjects

Subject	Age /Sex	Experiment	Electrodes location (Region of interest)	# of electrodes	Diagnosis
#1	40/M	Pin-point	L (S1 and S2)	48	TLE
#2	24/M	Pin-point	L (S2)	54	OLE
#3	36/F	Texture	R (S2)	72	TLE
#4	31/M	Pin-point & texture	R (S1)	84	TLE
#5	34/F	Pin-point & texture	R (S1)	92	PLE
#6	25/M	Pin-point (2 sessions) & texture	L (S1)	58	PLE/TLE

Abbreviation: F, female; M, male; R, right; L, left; PLE = parietal lobe epilepsy, OLE = occipital lobe epilepsy, TLE = temporal lobe epilepsy.
